# Product, building, and infrastructure material stocks dataset for 337 Chinese cities between 1978 and 2020

**DOI:** 10.1038/s41597-023-02143-w

**Published:** 2023-04-20

**Authors:** Xiang Li, Lulu Song, Qiance Liu, Xin Ouyang, Ting Mao, Haojie Lu, Litao Liu, Xiaojie Liu, Weiqiang Chen, Gang Liu

**Affiliations:** 1 Institute of Geographic Sciences and Natural Resources Research, Chinese Academy of Sciences, Beijing, 100101 China; 2 University of Chinese Academy of Sciences, Beijing, 100049 China; 3 Key Lab of Urban Environment and Health, Institute of Urban Environment, Chinese Academy of Sciences, 361021 Xiamen, Fujian China; 4 Xiamen Key Lab of Urban Metabolism, 361021 Xiamen, Fujian China; 5 Department of Green Technology, University of Southern Denmark, Odense, 5230 Denmark; 6 College of Urban and Environmental Sciences, Peking University, 100871 Beijing, China

**Keywords:** Sustainability, Socioeconomic scenarios

## Abstract

Reliable city-level product, building, and infrastructure material stocks data are essential for understanding historical material use patterns, benchmarking material efficiency, and informing future recycling potentials. However, such urban material stocks data are often limited, due primarily to unavailable, inconsistent, or noncontinuous city-level statistics. Here, we provided such an Urban Product, Building, and Infrastructure Material Stocks (UPBIMS) dataset for China, a country that has undergone a remarkable urbanization process in the past decades, by collating different official statistics and applying various gap-filling methods. This dataset contains the stock of 24 materials contained in 10 types of products, buildings, and infrastructure in all 337 prefecture-level cities in China from 1978 to 2020. This quality controlled and unified dataset is the first of its kind with such a full coverage of all prefecture-level Chinese cities and can be used in a variety of applications, for example in urban geography, industrial ecology, circular economy, and climate change mitigation. Every piece of data is tagged with its source and the dataset will be periodically updated.

## Background & Summary

Urbanization is one of the most important factors in human history that drives the global consumption of natural resources to an ever-high level. For example, the stocks of man-made materials were estimated to already overweigh all life on earth^1^, leading to profound changes in essential life-sustaining functions of the planet Earth. The United Nations Environment Programme (UNEP) warned that further urbanization and demand for urban services such as sheltering and mobility could raise the annual use of resources to nearly 90 billion tonnes by 2050, a 125% increase from 40 billion tonnes in 2010^2^. Sustaining long-term resource use and minimizing consequent environmental impacts accompanied by continuous urbanization requires thus a good understanding of how we have been accumulating materials in products, buildings, and infrastructure (so called in-use stocks) for urban services^3^. The spatiotemporal patterns of such stocks can help reveal the exchange, storage, and transformation of materials between the natural environment and cities and benchmark material efficiency^4^ and inform future resource demand, waste management challenges, and urban mining potentials^5–8^.

As the most populous and world’s second-largest economy, China is an ideal case for exploring the historical material stocks development pattern at city level^9^. The enormous rural-urban population migration and growth of cities in China in the past four decades have resulted in a profound expansion of urban built environment stocks^10,11^. For example, between 1990 and 2010, China’s share in global material stocks increased from 10% to 22%^12^. This continuously expanding urban resource demand further increases pressure on China’s already challenging resource, waste, and climate challenges^13,14^.

Generally, in-use material stocks can be estimated by either a top-down or a bottom-up approach^5^. The top-down method is often employed to evaluate the global and national material stocks by considering the differences between inflows (often available from industrial statistics) and outflows (estimated based on lifetime delay) of materials^15,16^. However, this is often challenging for material stock estimation on the regional or city levels due to the lack of material consumption (inflow) statistics. Instead, the bottom-up method is often used to estimate the urban material stocks by counting each item of products, buildings, and infrastructure and multiplying by their corresponding material intensity^4^. For China for example, a few studies have calculated China’s in-use material stocks at the national^17,18^ and provincial levels^18^, including with product (e.g., as infrastructure and household durable goods) and material (e.g., 24 types of materials considered in some studies^14,19,20^) resolution. There are also a few attempts on characterizing materials stocks for large cities (e.g., Beijing^21,22^, Chongqing^23^, and Xiamen^24^) in China; yet the overall city coverage is very low and medium and small cities are usually excluded. This relates mainly to data gaps on the city level caused by, e.g., inconsistent or noncontinuous local statistics and change of administrative areas. Considering there are 337 prefecture-level cities in China and they are at varying urbanization stages and have distinct developmental pathways^25^, it would be important to develop a complete dataset that covers all cities and years to reveal the spatiotemporal patterns of urban product, building, and infrastructure material stocks^20^.

In this study, we aim to provide such an Urban Product, Building, and Infrastructure Material Stocks (UPBIMS) dataset for China that have been compiled by collating urban material stock related statistical data from various yearbooks, bulletins, and agencies. Moreover, we have filled gaps for missing data (about 55% of all the data records) based on rational principles and consistent assumptions. Eventually, based on a bottom-up stock accounting method, we present a dataset on total weight and 24 types of material contained in 10 subtypes of products, buildings, and infrastructure (which are further categorized into residential buildings, nonresidential buildings, roads, urban rails, pipes, other infrastructure, vehicles, agricultural machinery, industrial machinery, and appliances) in active use in 337 cities of mainland China from 1978 to 2020.

The rest of the paper summarizes the accounting scopes, data sources, and gap filling approaches we used and the quality of this dataset. We will maintain and periodically update this dataset in the future. This comprehensive and consistent dataset of material stocks of all Chinese prefecture-level cities can help understand spatiotemporal patterns of urban weight growth, inform future materials demand associated with continuous urbanization, optimize construction and demolition waste management, facilitate discussion on embodied emissions of construction, and thus support the circular and low-carbon transition of cities in China and beyond.

## Methods

### Spatial and temporal boundary

Based on China’s administrative divisions, our dataset covered all 337 major cities in China, including four municipalities directly under the Central Government (i.e., Beijing, Shanghai, Tianjin, and Chongqing) and 333 prefecture-level cities belonging to 23 provinces and 5 autonomous regions. Their codes and changes of names in the past 43 years are available in sheet ‘Code’ in file ‘Data source.xlsx’ on Figshare^26^. Our spatial accounting scope is municipal districts and townships^23^. All these 337 cities host over 64% of China’s population and cover above 75% of built-up area in China. The time horizon of our dataset is from 1978 (when China started its reform and opening-up and thus cities started to grow) to 2020 (when the latest data are accessible). All computations were performed in time-discrete steps of one year.

### Material stocks of products, buildings, infrastructure

We used a bottom-up stock accounting method^15,27^ and counted all pieces of products, buildings, and infrastructure in cities over time to determine their embodied material stocks. The scope of urban product, building, and infrastructure stock items and corresponding estimation methods and data sources for their embodied material stocks are summarized in sheet ‘Scope’ in file ‘Data source.xlsx’ on Figshare^26^ and described in detail in the following sections.

We have attempted to include all products, buildings, and infrastructure in these cities. This adds up to 10 types of products, buildings, and infrastructure (details in sheet ‘Scope’ in file ‘Data source.xlsx’ on Figshare^26^). Based on significance of embodied materials and available material intensity data (detailed in file ‘Data source.xlsx’ and ‘Material intensity.xlsx’ on Figshare^26^), we considered 24 types of materials, including 13 types of base materials (steel, copper, aluminum, timber, brick, gravel, sand, asphalt, lime, glass, cement, plastic, and rubber), 3 types of precious metals (gold, silver, and palladium), 4 types of rare metals (indium, neodymium, yttrium, and europium), and 4 types of other metals (lead, zinc, magnesium, and cobalt).

### Data sources

The data on urban product, building, and infrastructure stock items are from official statistics or otherwise estimations based upon rational principles and consistent assumptions. A total of 1259 official statistical bulletins or yearbooks were compiled and used to derive data for population and products, buildings, and infrastructure (details in sheet ‘References’ in file ‘Data source.xlsx’ on Figshare^26^). These original statistical data, however, only directly report 45% of all possible stock data across city, time, and type of stock. In the following sections, we have described different sources and responsible departments of such statistics and how we have filled the data gaps systematically (details in sheet ‘Statistics’ in file ‘Data source.xlsx’ on Figshare^26^).

### Household survey data for buildings and appliances

We obtained the stock of urban residential buildings and home appliances by multiplying the per household (or per capita) stock and the numbers of family (or population). This is because per capita residential floor area (PC-RA) and the amount of home appliances per 100 urban households (PH-UA) are covered by the household survey of the National Bureau of Statistics of China (NBSC).

Such household survey was based upon a stratified multistage random sampling method. For example, in 2021, the NBSC selected about 160,000 urban households from 1,800 counties/districts across China. At the end of each quarter, the survey results are aggregated upward from the county level to the national level and published by the NBSC. This data summary method means that the PC-RA and PH-UA data are quarterly and county/district based. It should be noted that, after China introduced the urban-rural integration plan in 2013, the sampling scope accordingly expanded from municipal districts to municipal districts and townships in the urban-rural fringe. Nevertheless, the sample size of townships is much smaller than that of municipal districts, so that sampling scope change affected only 24% of the cities (with a decrease or increase of stock by more than 10%). Therefore, we have disregarded this scope change for the sake of consistency in statistical caliber.

These aggregated national and provincial data are annually published in the China Statistical Yearbook, China Household Survey Statistical Yearbook, and the National Statistical Data Release Database. However, it is often difficult to get the data for successive years at the municipal or city level, due to lack of strict rules on the frequency of data release. The local governments and statistical departments publish survey results only for selected years in their statistical bulletins and yearbooks.

It should be noted that there are no official data on nonresidential building floor areas in China. A few previous studies estimated the stock of nonresidential buildings simply the same as that of residential buildings^15,22,23^. We have improved this estimation by approximating the ratios of residential to nonresidential building floor areas of these cities based on their corresponding, and largely available, provincial ratios.

Due to data gaps and our focus on urban areas, the building stocks in rural areas within the urban municipal administrative boundaries are excluded from this analysis. The building stocks in rural areas did not experience a dramatic change comparing to urban building stocks. Indeed, it has grown less than 18% from 2000 to 2015^28^, and such a growth rate is further declining^29^ with the continuous urbanization.

### Ministerial statistics for infrastructure, machinery, and vehicles

Infrastructure, machinery, and vehicle stock data are collected and maintained by respective ministries in China, although not always with a consistent basis. For example, the stock of all vehicles (including passenger vehicles, trucks, and motorcycles) is tracked by the Ministry of Public Security (MPS) and their local branches. So, the vehicle stock data at the city/municipality levels are published by the local statistical bureau, while the NBSC publishes national and provincial aggregated data in the China Statistical Yearbook. We were able to gather data on vehicle stocks from the local statistical yearbooks and local statistical bulletins of all 337 cities.

Similarly, the Ministry of Housing and Urban-Rural Development (MOHURD) is in charge of the infrastructure data in China. The MOHURD publishes the end-of-year infrastructure stock data in the China Urban-Rural Construction Statistical Yearbook and China Urban Construction Statistical Yearbook at the city level since 2002, which includes data on roads, subways, pipelines, and street lamps. The data for previous years (1978–2001) can be obtained from the statistical bulletins and yearbooks published by local governments and the local statistical bureau.

Ministry of Agriculture and Rural Affairs (MARA) takes care of the administration and compilation of agricultural machinery data. The stock (installed capacity) of agricultural machinery can be obtained from the local statistical yearbooks and local statistical bulletins of all 337 cities. The stock of industrial machinery is unfortunately not available in any statistics. Instead, we approximated their installed capacity based on the stock of agricultural machinery and the total power consumption by agricultural and industrial machinery. This method has been applied before in quite a few similar studies due to the data unavailability of industrial machinery^23,24,30–32^. And most of them indicated high uncertainties but low sensitivity for this method^23^.

### Population and urbanization

The data on population and the number of families in the cities are key to our results, particularly for scaling up per capita residential floor areas and per 100 households appliances, and they were obtained from population surveys initiated by the NBSC and local governments. To estimate material stocks in municipal districts and townships, we chose to use the urban permanent population which refers to the actual population living there for over six months, as opposed to household registry population. This is also consistent with the sampling scope of household survey of the NBSC (source for our residential building and appliances data).

The permanent population data are from three types of population surveys in China. The first is the country-wide census, which is conducted every ten years. The data for those years ending as zero come from this census which can be obtained from the NBSC. The second is the sample survey of 1% of the national population, which is conducted every five years. The data for those years ending as five comes from this survey which can be obtained from the provincial bureau of statistics. The third is the national population change survey which is conducted every year except for the years when the first two types were conducted. The sample size accounts for about 1‰ of the total population. However, these permanent population data are not published consistently across cities. We filled in the missing data based on trend extrapolation between total urban permanent population and urbanization rate of cities. This is deemed acceptable because overall the missing share of total population in all 337 cities is only 11% (details in sheet ‘Statistics’ in file ‘Data source.xlsx’ on Figshare^26^). The number of families in the 337 cities was estimated based upon per household population from household survey.

### Data gap filling and estimation

**Fig. 1:**
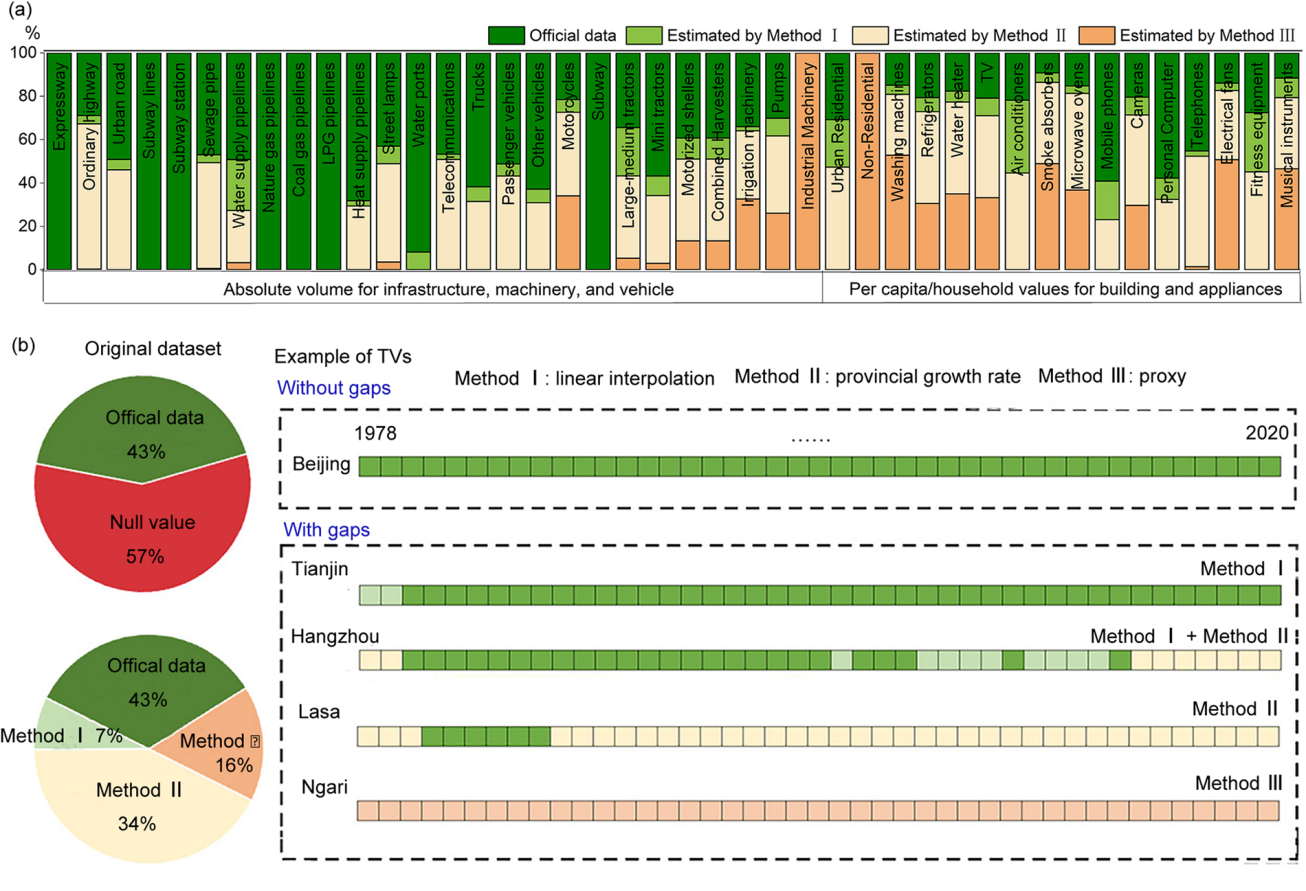
Patterns of missing data and corresponding gap filling methods: (**a**) the proportions of estimated data based on different gap filling methods by products, buildings, and infrastructure; and (**b**) shares and examples of data filling methods.

Significant amounts of missing values can be observed in the initially compiled database. This relates for example to either discontinuity in the data released by prefecture-level governments and statistical agencies, or the change of scope of urban administrative regions over time (see Fig. 1a). We have documented the original data source of each item used in the formula and calculated the missing ratio of all items in stock calculation functions in ‘Data sources.xlsx’ on Figshare^26^. Depending on the patterns and proportions of missing values of these items across 43 years (from 1978 to 2020) in 337 cities, three different methods were used either individually or in combination (see Fig. 1b) to fill the gaps:

- Method I estimation is based partially on linear interpolation. We used this method to fill data gaps for those series with a missing rate below 10% or missing data between two years. This 10% is set based on the statistical principle^33^ that if the missing rate is more significant than 10%, the result of subsequent statistical analyses may be biased.
- Method II estimation is based partially on the provincial stock growth rate. We used this method to fill gaps for those series with a missing rate above 10%. This is particularly the case for continuously missing values before the starting year or after the ending year with values in the series. The provincial stock growth rate, which was derived from available statistics on the provincial level, was applied to the city level to estimate these missing values.
- Method III estimation is based entirely on proxy. We used this method to fill gaps where there are no city-level data mainly for three types of issues. The first refers to missing household survey values for some cities in recent years; and we assumed them the same as the provincial averages. The second type refers to missing absolute volumes for some cities; and we approximated them by multiplying provincial data with these cities’ share of GDP in their provinces. The third type relates to the conversion ratio assumptions among products, as we elaborated above for nonresidential buildings (based on residential buildings and land use) and industrial machinery (based on agricultural machinery).

In the end, we have successfully filled all the data gaps that initially account for approximately 55% of all data points. Although 17% of the data were populated using Method III (proxy) alone, more than 50% out of these proxy data were based actually on household surveys (details in sheet ‘Statistics’ in file ‘Data source.xlsx’ on Figshare^26^). This ratio means that most of the missing data is filled in on the basis of some sort of official statistics. Hence, our eventually imputed dataset is deemed as reasonably accurate to reveal the overall spatiotemporal patterns of product, building, and infrastructure material stocks among cities.

### Material intensity

The material intensities (MI) of the 24 types of selected materials were compiled from literature^14,19,23,32,34–47^. Due to limited primary MI data, quite a few studies refer to the same sources for MI estimation; and in such cases, we only listed those references that first reported these MI data. The MI data were described in detail in our open-access online dataset Figshare^26^.

## Data Records

This dataset contains three core tables, which are all provided as xlsx files and are freely available through Figshare^26^. A total of 1,741,680 data records are contained in the dataset. Of these,

- 159,401 records are weight in total and by 11 types of materials embodied in urban residential buildings in 337 cities from 1978 to 2020 [‘*Urban residential building stocks 1978*–*2020.xlsx*’ on Figshare^26^];
- 159,401 records are weight in total and by 11 types of materials embodied in urban nonresidential buildings in 337 cities from 1978 to 2020 [‘*Urban nonresidential building stocks 1978*–*2020.xlsx*’ on Figshare^26^];
- 101,437 records are weight in total and by 7 types of materials embodied in roads in 337 cities from 1978 to 2020 [‘*Road stocks 1978*–*2020.xlsx*’ on Figshare^26^];
- 115,928 records are weight in total and by 8 types of materials embodied in urban rails in 337 cities from 1978 to 2020 [‘*Urban rail stocks 1978*–*2020.xlsx*’ on Figshare^26^];
- 86,946 records are weight in total and by 6 types of materials embodied in pipelines in 337 cities from 1978 to 2020 [‘*Pipeline stocks 1978*–*2020.xlsx*’ on Figshare^26^];
- 43,473 records are weight in total and by 3 types of materials embodied in other infrastructure (street lamps and telecommunications) in 337 cities from 1978 to 2020 [‘*Other infrastructure stocks 1978*–*2020.xlsx*’ on Figshare^26^];
- 144,910 records are weight in total and by 10 types of materials embodied in vehicles in 337 cities from 1978 to 2020 [‘*Vehicle stocks 1978*–*2020.xlsx*’ on Figshare^26^];
- 43,473 records are weight in total and by 3 types of materials embodied in agricultural machinery in 337 cities from 1978 to 2020 [‘*Agricultural machinery stocks 1978*–*2020.xlsx*’ on Figshare^26^];
- 14,491 records are weight in total and by 1 type of materials embodied in industrial machinery in 337 cities from 1978 to 2020 [‘*Industrial machinery stocks 1978*–*2020.xlsx*’ on Figshare^26^];
- 188,383 records are weight in total and by 13 types of materials embodied in appliances in 337 cities from 1978 to 2020 [‘*Appliance stocks 1978*–*2020.xlsx*’ on Figshare^26^];
- 57,964 records are total material stocks, per capita material stocks, urban populations, and household numbers of 337 cities from 1978 to 2020 [‘*Material stock and population 1978*–*2020.xlsx*’ on Figshare^26^];
- 654 records are material intensity data and corresponding sources for the 24 materials embodied in 42 types of products, buildings, and infrastructure [‘*Material intensity.xlsx’* on Figshare^26^];
- 625,219 records are the original data sources for the population and 42 types of products, buildings, and infrastructure items in the 337 cities from 1978 to 2020 [‘*Data source.xlsx*’ on Figshare^26^].

## Technical Validation

### Data overview

**Fig. 2:**
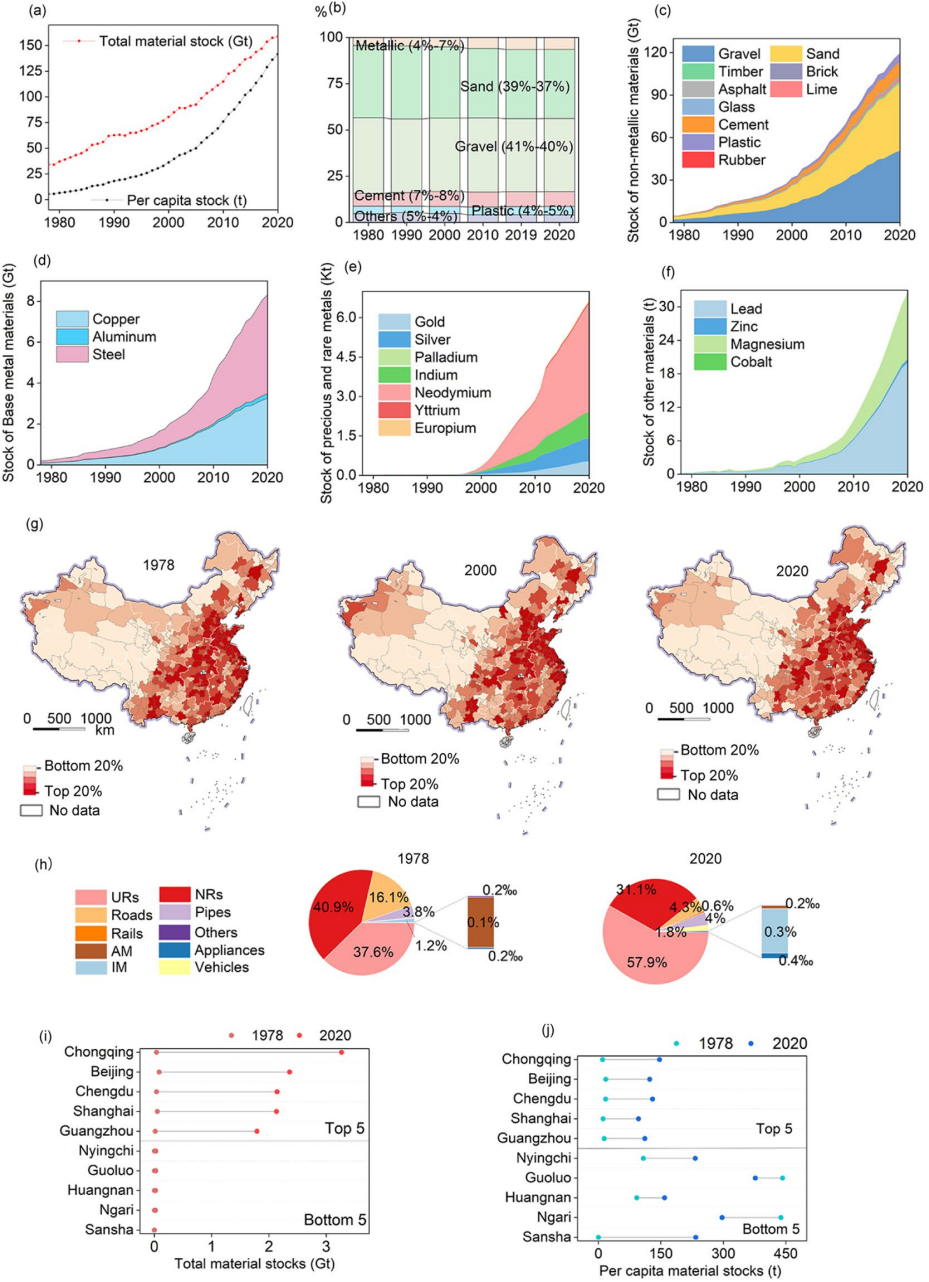
Spatio-temporal patterns of urban material stocks in 337 Chinese cities during 1978–2020: (**a**) The total and per capita material stocks of all cities; (**b**) Temporal stock patterns by materials of all cities; (**c**) Total stocks of non-metallic materials; (**d**) Total stocks of base metals; (**e**) Total stocks of precious and rare metals; (**f**) Total stocks of other metals; (**g**) Spatial distribution of urban material stocks by cities in 1978, 2000, and 2020; (**h**) Temporal stock patterns by end use sectors of all cities; (**i**) The total material stocks of the top five cities and bottom five cities in 1978 and 2020; and (**j**) The per capita material stocks of the top five cities and bottom five cities in 1978 and 2020. (URs: urban residential buildings; NRs: nonresidential buildings; AM: agricultural machinery; IM: industrial machinery).

The material stocks in Chinese cities have steadily increased during 1978–2020, especially in the early 21st century, when the growth rate peaked (Fig. 2a). Material wise, non-metallic materials used in buildings and infrastructure such as sand (39%-37%) and gravel (41%-40%) take up the largest portion among all materials over time due to their bulky nature and large volume of use in construction (Fig. 2b). The growth rate of these materials (Fig. 2c), however, has slowed since 2015 when China’s infrastructure boom started to level off (see the decreased proportion of roads and non-residential buildings from 1978 to 2020 in Fig. 2h). In contrast, the growth of metal stocks has shown a steadily increasing trend (Fig. 2d–f) with the increasing ownership of buildings, appliances, and vehicles in urban households (see an overview in Fig. 2h and more details on Figshare^26^).

**Fig. 3:**
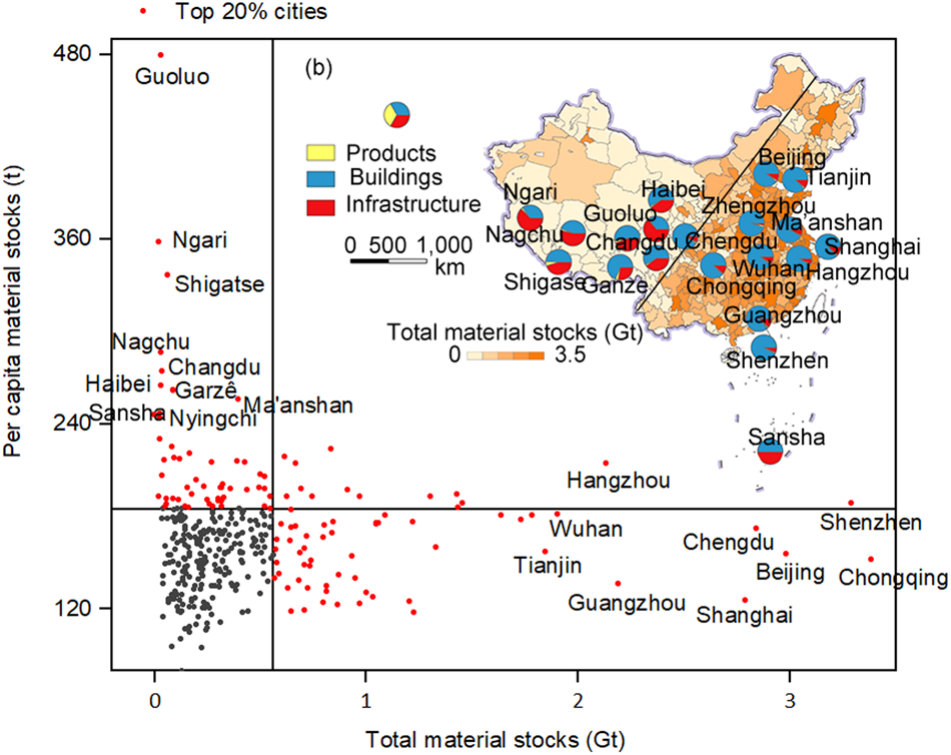
The spatial and sectoral patterns of urban material stocks of 337 Chinese cities in 2020: (**a**) The comparison between per capita and total material stocks of all cities, with the top 20% highlighted in red; and (**b**) The total material in-use stocks and shares in products, buildings, and infrastructure of the top 10 and bottom 10 cities.

Regionally, China’ urban material stock shows an uneven geographical distribution between cities in the southeast and in the northwest, which is in accordance with China’s uneven pattern of urbanization, as indicated by the Hu Line (that is, the “Aihui-Tengchong” line which is a famous dividing line of China’s climate, population, economic, and social patterns between the southeast and northwest, see more details in Fig. 2g and sheet ‘Code’ in file ‘Data source.xlsx’ on Figshare^26^)^48,49^. Cities in eastern China have been always among the top across the country in terms of urban material stocks from 1978 to 2020, and the difference between eastern and western cities has widened (Fig. 2h). The total material stocks and per-capita material stocks of all 337 cities have been growing in the past 40 years. Although the difference among total material stocks of the 337 cities is tremendous (Fig. 2i), per capita material stocks of different cities appears comparably more balanced (Fig. 2j). The growth rate of per capita stocks in small cities with low stock is equal to or even higher than that in large cities with high stock. For example, Beijing’s total urban material stock growth between 1978 and 2020 was 176 times that of Ngari. Per-capita stocks in Ngari is 297 t in 2020, which is twice of Beijing’s 123 t (see Fig. 3g,f for more details in ‘Material stock and population 1978–2020.xlsx’ on Figshare^26^).

It is noteworthy that the spatial distribution of per capita urban material stocks is different from that of the total stock. Although approximately 95% of urban total material stocks in 2020 were accumulated on the east of the Hu line^49^, many cities in the west have actually higher per capita stocks. This contrast reflects the varying composition of in-use products. For example, the top 10 cities with the highest proportion of infrastructure stocks are distributed on the sparsely populated west of the Hu line, and have very high per capita stocks; while in contrast, most densely populated cities in the east have a higher share of stock in buildings (see Fig. 3 for an overview and more details on Figshare^26^). This also indicates the scale of economy and higher efficiency of materials use for service provision in denser and more developed cities.

### Comparison with existing estimates

When our stock estimates are compared with literature values (Table 1), the material stocks results are overall close to studies based on similar research methods. We want to highlight below two major differences between our scope and data sources and that of previous studies.

**Table 1:** Comparison of our urban material stocks estimation with literature values.

Region/City	Reference	Method	Year of estimation	Accounting items		Scope	Earlier estimates	This study	Difference
China	Yue *et al*.^52^	Top-down	2009	Products, infrastructure, and buildings	Aluminum	Urban and rural	88.9 Mt	71 Mt	20%
	Yue *et al*.^52^	Top-down	2009	Products, infrastructure, and buildings	Copper	Urban and rural	51.4 Mt	32 Mt	38%
	Krausmann *et al*.^12^	Top-down	2010	Products, infrastructure, and buildings	Total stock	Urban and rural	181 Gt	56.7 Gt	69%
	Song *et al*.^32^	Bottom-up	2010	Buildings	steel	Urban and rural	2.1 Gt	1.2 Mt	43%
	Sun *et al*.^53^	Bottom-up	2016	Appliances	Plastic	Urban and rural	24 Mt	12.4 Mt	48%
Beijing	Fu *et al*.^21^	Bottom-up	2015	Products, infrastructure, and buildings	Total stock	Urban and rural	1925 Mt	2071 Mt	−8%
	Mao *et al*.^35^	Bottom-up and Geographical Information System	2018	Building	Total stock	Urban	2270 Mt	2038 Mt	10%
	Song *et al*.^32^	Bottom-up	2018	Products, infrastructure, and buildings	Total stock	Urban and rural	2588 Mt	2358 Mt	9%
Shanghai	Zhang *et al*.^31^	Top-down & Bottom-up	2012	Products, infrastructure, and buildings	copper	Urban	0.9 Mt	1.2 Mt	−33%
	Song *et al*.^32^	Bottom-up	2018	Products, infrastructure, and buildings	Total stock	Urban and rural	4475 Mt	2433 Mt	46%
Tianjin	Liu *et al*.^23^	Bottom-up	2016	Products, infrastructure, and buildings	Aluminum	Urban	2.9 Mt	2.3 Mt	21%
	Song *et al*.^32^	Bottom-up	2018	Products, infrastructure, and buildings	Total stock	Urban and rural	2384 Mt	1267 Mt	47%
Nanjing	Zhang *et al*.^30^	Bottom-up	2009	Products, infrastructure, and buildings	copper	Urban	0.30 Mt	0.31 Mt	−3%
	Yu, B. *et al*.^37^	Remote Sensing	2020	Roads	Total stock	Urban and rural	42.9 Mt	20.3 Mt	53%
Xiamen	Song *et al*.^24^	Bottom-up	2015	Products, infrastructure, and buildings	steel	Urban and rural	13 Mt	14.8 Mt	−14%

First, the total stock of some materials (e.g., gold^14^, steel^34^, and plastic^35^) appears moderately different from that of previous studies on the country level, due mainly to scope differences. Our spatial scope is limited to the urban area, and we have not considered the rural buildings and rural appliances. Meanwhile, we have included fewer items of products, buildings, and infrastructure in stock accounting, as our much wider city coverage does not allow for data collection for all cities, e.g., railways, water and environmental infrastructures, and appliances for commercial use.

Second, we explicitly considered the household survey and population statistical scope when estimating the building and appliance stocks, and used the permanent population (in line with the sampling scope of the urban household survey) to estimate the total floor area and household appliances instead of the household registry population. Therefore, residential building stocks in our dataset are generally higher than in previous bottom-up studies (see the examples of Beijing^39^ and Shanghai^50^).

Overall, despite some of our research scope limitations, our quality controlled and unified dataset is deemed reliable and is actually the first of its kind with such a full coverage of all prefecture-level Chinese cities, which can thus be further used in a variety of applications, for example in urban geography, circular economy, and climate change mitigation research.

### Limitations and uncertainties

Despite our best efforts, our datasets bear some inevitable uncertainties and limitations, which should be pointed out as below and remain to be addressed in the future to improve the accuracy.

- First, due to data paucity, 55% of the data is estimated by data gap-filling methods. Although the assumptions for gap filling were based on reliable official data and justified principles, this scale of data filling would unavoidably result in systemic errors in the imputed database. This can be better validated and cross-checked by more independent estimation based on other methods in the future.
- Second, the material intensities derived from literature come with high uncertainty, due to unfortunately very little information on this regard, not to mention region specific differences. For example, the material intensity may vary by time (while we only considered the material intensity changes for a few products such as TVs, computers, and buildings) and by size and brands of products (which we didn’t consider at all). Although this is a common limitation in most previous bottom-up material stock studies^32^, it would be beneficial to establish more community-wide and reliable material inventory databases for key products and infrastructure in future research, as exemplified for buildings^19,20^.
- Third, the various sources of statistics from different yearbooks, bulletins, and agencies bear uncertainty as well. For example, the household survey data used in the calculation are obtained from the NBSC survey on 1% of the population and does not fully cover all urban residents, and the population statistical scope changes over time as well as we elaborated in the Method section. This uncertainty may lead to shrinking building stocks in some cities as their population decreases (e.g., cities in northeast China like Siping).
- Fourth, our research scope is limited to urban areas, while stocks of some products such as agricultural machinery and industrial machinery could not be split between urban and rural explicitly; and stock of industrial machinery is approximated by agricultural machinery, thus with high uncertainty. Nevertheless, we can confirm that agricultural and industrial machinery together accounted for only a small proportion (below 0.4% in 2020) of the total material stocks, so this would not affect our overall results that much.

**Fig. 4:**
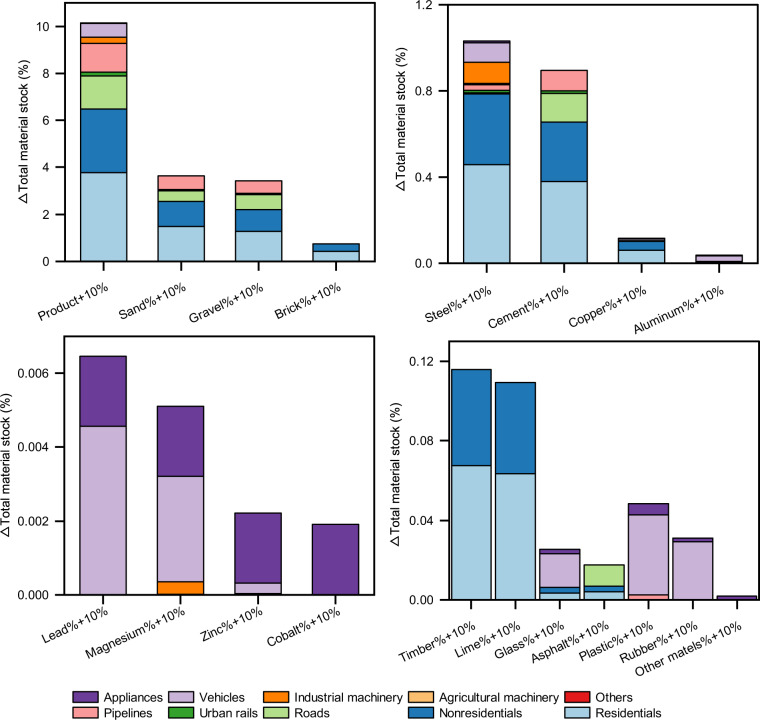
Sensitivity analysis results for the year 2020 average stock of all 337 cities by increasing product quantity or material intensity by 10%.

To assess the sensitivity and compare the impact of each parameter (i.e., product quantity and material intensity), we conducted a one-at-a-time sensitivity analysis (e.g., by increasing one parameter by 10% while keeping others unchanged) to examine the effect of variation of parameters separately^51^. Figure 4 shows the sensitivity analysis results for the average stocks of the 337 cities in the year 2020, and results across all 43 years and other details are available in sheet ‘Sensitivity’ in file ‘Data source.xlsx’ on Figshare^26^. We can see that among product quantities, building floor areas have the largest impact on the total material stocks, while for material intensities, most materials have a negligible impact on total material stock (less than 1%), except for sand and gravel (above 3%).

All these uncertainties and limitations should be paid special attention when this dataset is used and interpreted. We also aim to address them in the future, e.g., by expanding the product and materials selection and improving the material intensity estimation. This data structure could be extended to even smaller cities below the prefecture-level (i.e., county level) as well. Since every piece of data is tagged with its source in the dataset, we plan to periodically update this dataset.

## Usage Notes

The data files are documented as xlsx files, which can be readily read and processed by many software, such as Matlab, R, and Python. The 24 materials can be segmented into base materials, precious metals, rare metals, and other metals. Material intensity in ‘Material intensity 1978–2020.xlsx’ on Figshare^26^ detailed records of their accounting scope. Meanwhile, data sources are provided for each data record and our assumptions for estimated data are explicitly explained, so our estimates can be changed or excluded whenever needed. In addition, the data filling code provided in this study can also be used to fill other types of statistical data, depending on the applicable principles.

## Data Availability

The data gap were filled in Python 3.8, using the following libraries: pandas 1.3.4, numpy 1.18.5, statsmodels 0.13.2, sklearn 1.0.2, scipy 1.7.3. The full custom Python script is provided on the open-access online dataset Figshare^26^ [*‘Estimated code.ipynb’*].

## References

[1] Elhacham E, Ben-Uri L, Grozovski J, Bar-On YM, Milo R (2020). Global human-made mass exceeds all living biomass. Nature.

[2] IRP. *The weight of cities: resource requirements of future urbanization*. (2018).

[3] Tan LM, Arbabi H, Densley Tingley D, Brockway PE, Mayfield M (2021). 2–20 Mapping resource effectiveness across urban systems. npj Urban Sustainability.

[4] Chen WQ, Graedel TE (2015). In-use product stocks link manufactured capital to natural capital. Proc Natl Acad Sci USA.

[5] Lanau, M., Liu, G., Kral, U., Wiedenhofer, D., Keijzer, E., Yu, C., & Ehlert, C. Taking Stock of Built Environment Stock Studies: Progress and Prospects. *Environ Sci Technol* **53**, 8499–8515 (2019). 10.1021/acs.est.8b06652 31246441

[6] Aguilar-Hernandez GA, Deetman S, Merciai S, Rodrigues JFD, Tukker A (2021). Global distribution of material inflows to in-use stocks in 2011 and its implications for a circularity transition. J Ind Ecol.

[7] Kuong IH, Li J, Zhang J, Zeng X (2019). Estimating the Evolution of Urban Mining Resources in Hong Kong, Up to the Year 2050. Environ Sci Technol.

[8] Bauer S (2012). Sustainable materials: With both eyes open. Materials Today.

[9] Xu M, Zhang T, Allenby B (2008). How much will China weigh? Perspectives from consumption structure and technology development. Environ Sci Technol.

[10] Dai K, Shen S, Cheng C (2022). Evaluation and analysis of the projected population of China. Sci Rep.

[11] Zhang KH, Song S (2003). Rural-urban migration and urbanization in China: Evidence from time-series and cross-section analyses. China Economic Review.

[12] Krausmann F (2017). Global socioeconomic material stocks rise 23-fold over the 20th century and require half of annual resource use. Proc Natl Acad Sci USA.

[13] Mathews JA, Tan H (2016). Circular economy: Lessons from China. Nature.

[14] Zeng X, Ali SH, Tian J, Li J (2020). Mapping anthropogenic mineral generation in China and its implications for a circular economy. Nat Commun.

[15] Tanikawa H, Fishman T, Okuoka K, Sugimoto K (2015). The weight of society over time and space: A comprehensive account of the construction material stock of Japan, 1945–2010. J Ind Ecol.

[16] Müller E, Hilty LM, Widmer R, Schluep M, Faulstich M (2014). Modeling metal stocks and flows: A review of dynamic material flow analysis methods. Environ Sci Technol.

[17] Zeng X, Ali SH, Li J (2021). Estimation of waste outflows for multiple product types in China from 2010–2050. Sci Data.

[18] Wiedenhofer, D. *et al*. Prospects for a saturation of humanity’s resource use? An analysis of material stocks and flows in nine world regions from 1900 to 2035. *Global Environmental Change* **71** (2021).

[19] Heeren, N. & Fishman, T. A database seed for a community-driven material intensity research platform. *Sci Data* **6** (2019). 10.1038/s41597-019-0021-x PMC6480936 30967550

[20] Sprecher B (2022). Material intensity database for the Dutch building stock: Towards Big Data in material stock analysis. J Ind Ecol.

[21] Fu C, Zhang Y, Yu X (2019). How has Beijing’s urban weight and composition changed with socioeconomic development?. Science of the Total Environment.

[22] Mao R, Bao Y, Huang Z, Liu Q, Liu G (2020). High-Resolution Mapping of the Urban Built Environment Stocks in Beijing. Environ Sci Technol.

[23] Liu Q (2019). Product and Metal Stocks Accumulation of China’s Megacities: Patterns, Drivers, and Implications. Environ Sci Technol.

[24] Song L, Zhang C, Han J, Chen WQ (2019). In-use product and steel stocks sustaining the urbanization of Xiamen, China. Ecosystem Health and Sustainability.

[25] Shan Y (2018). City-level climate change mitigation in China. Sci Adv.

[26] Xiang L (2023). figshare.

[27] Gerst, M. D. & Graedel, T. E. In-use stocks of metals: Status and implications. *Environ Sci Technol* **42**, 7038–7045 (2008). 10.1021/es800420p 18939524

[28] Huo T (2019). China’s building stock estimation and energy intensity analysis. J Clean Prod.

[29] Han, F. *et al*. In-use stocks dynamic of durable goods in rural Chinese households: Spatial-temporal patterns and influencing factors. *Resour Conserv Recycl* **186** (2022).

[30] Zhang L, Yuan Z, Bi J (2012). Estimation of Copper In-use Stocks in Nanjing, China. J Ind Ecol.

[31] Zhang L, Cai Z, Yang J, Chen Y, Yuan Z (2014). Quantification and spatial characterization of in-use copper stocks in Shanghai. Resour Conserv Recycl.

[32] Song L (2021). China material stocks and flows account for 1978–2018. Sci Data.

[33] Bennett DA (2001). How can I deal with missing data in my study?. Aust N Z J Public Health.

[34] Wang T, Müller DB, Hashimoto S (2015). The ferrous find counting iron and steel stocks in China’s economy. J Ind Ecol.

[35] Zhang L, Yang J, Cai Z, Yuan Z (2015). Understanding the spatial and temporal patterns of copper in-use stocks in China. Environ Sci Technol.

[36] Mao R, Bao Y, Duan H, Liu G (2021). Global urban subway development, construction material stocks, and embodied carbon emissions. Humanit Soc Sci Commun.

[37] Yu, B. *et al*. Material stock quantification and environmental impact analysis of urban road systems. *Transp Res D Transp Environ* **93** (2021).

[38] Khumvongsa K, Guo J, Theepharaksapan S, Shirakawa H, Tanikawa H (2023). Uncovering urban transportation infrastructure expansion and sustainability challenge in Bangkok: Insights from a material stock perspective. J Ind Ecol.

[39] Li Y, Zhang Y, Yu X (2019). Urban weight and its driving forces: A case study of Beijing. Science of the Total Environment.

[40] Huang T, Shi F, Tanikawa H, Fei J, Han J (2013). Materials demand and environmental impact of buildings construction and demolition in China based on dynamic material flow analysis. Resour Conserv Recycl.

[41] Han J, Xiang WN (2013). Analysis of material stock accumulation in China’s infrastructure and its regional disparity. Sustain Sci.

[42] Department of Resource Conservation and Environmental Protection. *National Development and Reform Commission. Disposal of Waste Electrical and Electronic Products*. (Social Sciences Literature Press, 2013).

[43] Lewis, G. M., Buchanan C. A., Jhaveri, K. D., Sullivan, J. L., Kelly, J. C., Das, S., Taub, A. I. & Keoleian, G. A. Green Principles for Vehicle Lightweighting. *Environ Sci Technol* **53**, 4063–4077 (2019). 10.1021/acs.est.8b05897 30892881

[44] Serrenho AC, Allwood JM (2016). Material Stock Demographics: Cars in Great Britain. Environ Sci Technol.

[45] Christian B, Romanov A, Romanova I, Turbini LJ (2014). Elemental compositions of over 80 cell phones. J Electron Mater.

[46] Singh N, Duan H, Yin F, Song Q, Li J (2018). Characterizing the Materials Composition and Recovery Potential from Waste Mobile Phones: A Comparative Evaluation of Cellular and Smart Phones. ACS Sustain Chem Eng.

[47] Hao M (2020). Spatial distribution of copper in-use stocks and flows in China: 1978–2016. J Clean Prod.

[48] Chen, D. *et al*. Exploring the spatial differentiation of urbanization on two sides of the Hu Huanyong Line – based on nighttime light data and cellular automata. *Applied Geography* **112**, (2019).

[49] Hu H (1935). The distribution of population in China. Acta Geographica Sinica.

[50] Han, J., Chen, W.-Q., Zhang, L., Liu, G. & Key, S. *Supporting Information Uncovering the spatiotemporal dynamics of urban infrastructure development: A high spatial resolution materials stock and flow analysis*. http://bbs.local.163.com. 10.1021/acs.est.8b03111 30277072

[51] Hughes M, Palmer J, Cheng V, Shipworth D (2013). Sensitivity and uncertainty analysis of England’s housing energy model. Building Research and Information.

[52] Yue Q, Wang HM, Lu ZW (2012). Quantitative estimation of social stock for metals Al and Cu in China. Transactions of Nonferrous Metals Society of China (English Edition).

[53] Sun N (2022). Material Flow analysis of plastics from provincial household appliances in China: 1978–2016. Waste Management.

